# Immunoglobulin G4 (IgG4)-Related Lung Disease Mimicking Pulmonary Malignancy: A Case Report With Histopathological Characterisation and Review of Diagnostic Criteria

**DOI:** 10.7759/cureus.115492

**Published:** 2026-08-31

**Authors:** Sant Das, Masood Shaikh, Sadhna Priya, Sana Khan

**Affiliations:** 1 Internal Medicine, Diana Princess of Wales Hospital, Grimsby, GBR; 2 Pulmonology, Queen Elizabeth Hospital, King's Lynn, King's Lynn, GBR; 3 Acute Medicine, Diana Princess of Wales Hospital, Grimsby, GBR; 4 Medicine, Aga Khan University Hospital, Karachi, PAK

**Keywords:** corticosteroids, igg4-related disease, igg4-related lung disease, immunosuppressive therapy, pet-ct, pulmonary malignancy mimicry, pulmonary pseudotumor, storiform fibrosis, thoracic oncology

## Abstract

Immunoglobulin G4 (IgG4)-related disease (IgG4-RD) is a systemic fibroinflammatory condition with highly variable pulmonary manifestations that frequently overlaps clinically, radiologically, and metabolically with primary malignancy, granulomatous disease, and vasculitis. Tissue diagnosis with immunohistochemical quantification of IgG4-positive plasma cells remains the diagnostic cornerstone.

A 50-year-old male ex-smoker presented with a two-month history of progressive cough, dyspnoea, chest pain, and 8 kg of unintentional weight loss. Examination revealed digital clubbing and bilateral crepitations. Laboratory investigations showed mild normocytic anaemia, leukocytosis with neutrophilic predominance, and a markedly elevated erythrocyte sedimentation rate of 109 mm/h. Serum IgG4 was elevated at 2.23 g/L (reference range <1.35 g/L). Total IgG and comprehensive autoimmune and infectious serology were unremarkable. Positron Emission Tomography-Computed Tomography (PET-CT) demonstrated multiple bilateral pulmonary nodules (some cavitating) with an exceptionally high maximal standardised uptake value (SUVmax 20.1) and mediastinal lymphadenopathy, prompting strong initial suspicion of primary pulmonary malignancy. CT-guided lung biopsy revealed dense storiform fibrosis, a lymphoplasmacytic infiltrate rich in eosinophils, and features of fibroinflammatory change; however, immunohistochemistry did not demonstrate an increased number of IgG4-positive plasma cells, and formal histopathological criteria for IgG4-related disease were not fulfilled. In the context of compatible clinical, radiological, and serological findings, a diagnosis of probable IgG4-related lung disease was considered. Oral prednisolone at 0.6 mg/kg/day produced substantial clinical and radiological improvement, with near-complete resolution of opacities and reduction in nodule size by six months.

This case illustrates the diagnostic pitfalls of IgG4-related lung disease, which can produce exceptionally high 18F-fluorodeoxyglucose (FDG) avidity on PET-CT, cavitating nodules, and constitutional features indistinguishable from malignancy. Histopathological evaluation with formal IgG4 cell quantification and ratio calculation is essential for accurate diagnosis; however, cases with supportive clinicoradiological features but non-diagnostic immunostaining may still pose significant diagnostic challenges. Early corticosteroid treatment yields excellent outcomes and prevents unnecessary oncological intervention.

## Introduction

Immunoglobulin G4 (IgG4)-related disease (IgG4-RD) is a systemic immune-mediated fibroinflammatory condition characterised by a pathological triad of storiform fibrosis, obliterative phlebitis, and dense lymphoplasmacytic infiltration enriched with IgG4-positive plasma cells. First recognised as a distinct clinicopathological entity in 2003 following observations in autoimmune pancreatitis, IgG4-RD is now understood to affect virtually any organ system, most commonly the pancreas, salivary glands, biliary tree, kidneys, and retroperitoneum [1]. IgG4 is one of four subclasses of immunoglobulin G and normally constitutes only a small proportion (approximately 3-6%) of total circulating IgG, contributing physiologically to immune tolerance under conditions of chronic antigen exposure. In IgG4-RD, dysregulated class-switching and expansion of IgG4-secreting plasmablasts are thought to underlie the tissue infiltration and fibrosis that characterise the disease, although the precise pathogenic contribution of the IgG4 antibody itself remains incompletely understood [2].

Pulmonary involvement occurs in approximately 20-30% of patients with IgG4-RD and encompasses a broad radiological spectrum including solid nodules, mass-like consolidations, ground-glass opacities, interstitial lung disease patterns, and mediastinal lymphadenopathy [3,4]. Because IgG4-related lung disease (IgG4-RLD) lacks pathognomonic clinical or radiological features, it poses a substantial diagnostic challenge, frequently mimicking primary pulmonary malignancy, granulomatosis with polyangiitis (GPA), and cryptogenic organising pneumonia (COP). The 2019 American College of Rheumatology and the European Alliance of Associations for Rheumatology (ACR/EULAR) classification criteria for IgG4-RD and the established histopathological consensus guidelines provide the current diagnostic framework, but both require tissue confirmation as their cornerstone [5,6].

We report a case of probable IgG4-related lung disease presenting with an exceptionally high Positron Emission Tomography-Computed Tomography (PET-CT) metabolic signal, cavitating pulmonary nodules, and constitutional features that prompted strong initial suspicion of primary lung malignancy. We discuss the histopathological diagnostic criteria, the limitations of PET-CT in distinguishing IgG4-RD from malignancy, and the atypical clinical features in this case that merit specific clinicopathological discussion. The classification and histopathological consensus criteria introduced above are revisited explicitly against this patient's findings in the Case Presentation and Discussion sections below, allowing direct comparison of which criteria were, and were not, met.

## Case presentation

Patient background

A 50-year-old man was referred with a two-month history of progressive productive cough, exertional dyspnoea, pleuritic chest pain, and unintentional weight loss of approximately 8 kg. He was a former smoker with a 20 pack-year history, having stopped 10 years prior. He had no significant comorbidities, was not taking regular medications, and had no occupational exposures to asbestos, silica, or other inorganic dusts. There was no family history of malignancy or autoimmune disease. A systematic review of symptoms did not identify sicca features, orbital swelling, jaundice, or urinary symptoms to suggest alternative sites of IgG4-related disease involvement.

Physical examination

Examination revealed digital clubbing (grade 2) and bilateral mid-to-upper zone end-inspiratory crepitations. There was no palpable lymphadenopathy, salivary gland enlargement, orbital proptosis, or abdominal organomegaly. He was haemodynamically stable with normal oxygen saturation on room air.

Laboratory investigations

Full blood count demonstrated mild normocytic anaemia (haemoglobin 120 g/L) and leukocytosis (16.2 × 109/L) with neutrophilic predominance. Absolute eosinophil count was not recorded. Erythrocyte sedimentation rate was markedly elevated at 109 mm/h. Serum IgG4 was elevated at 2.23 g/L (reference range <1.35 g/L; 1.65× upper limit of normal). Total serum IgG was within normal limits at 10.3 g/L. Comprehensive autoimmune serology including antinuclear antibody (ANA), rheumatoid factor, antineutrophil cytoplasmic antibody (ANCA) (proteinase 3 (PR3) and myeloperoxidase (MPO)), complement levels, and cryoglobulins was negative. Serum angiotensin-converting enzyme was within normal limits. Sputum cultures for bacterial pathogens, mycobacteria, and fungi were negative. HIV serology was negative. Renal and hepatic function panels were within normal limits. Urinalysis showed no proteinuria or haematuria. The autoimmune, infectious, and biochemical profile is summarised in Table 1.

**Table 1:** Summary of key laboratory investigations ESR: Erythrocyte sedimentation rate; CRP: C-reactive protein; IgG: Immunoglobulin G; ANA: Antinuclear antibody; ANCA: Antineutrophil cytoplasmic antibody; PR3: Proteinase 3; MPO: myeloperoxidase; ACE: Angiotensin-converting enzyme; HIV: Human immunodeficiency virus; AFB: Acid-fast bacilli.

Investigation	Result	Reference Range
Haemoglobin	120 g/L	130–170 g/L
White cell count	16.2 × 10⁹/L	4.0–11.0 × 10⁹/L
Differential: neutrophil predominance	Yes	N/A
Absolute eosinophil count	Not recorded	0.05–0.5 × 10⁹/L
ESR	109 mm/h	<20 mm/h
CRP	Not recorded	<5 mg/L
Serum IgG4	2.23 g/L	<1.35 g/L
Total serum IgG	10.3 g/L	7.0–16.0 g/L
ANA	Negative	N/A
Rheumatoid factor	Negative	N/A
ANCA (PR3 and MPO)	Negative	N/A
Serum ACE	Normal	<52 U/L
Renal/hepatic function	Normal	N/A
Urinalysis	Normal	N/A
HIV serology	Negative	N/A
Sputum cultures (bacterial, AFB, fungal)	Negative	N/A

Imaging

Chest radiography demonstrated ill-defined bilateral subpleural opacities with no pleural effusion (Figure 1).

**Figure 1:**
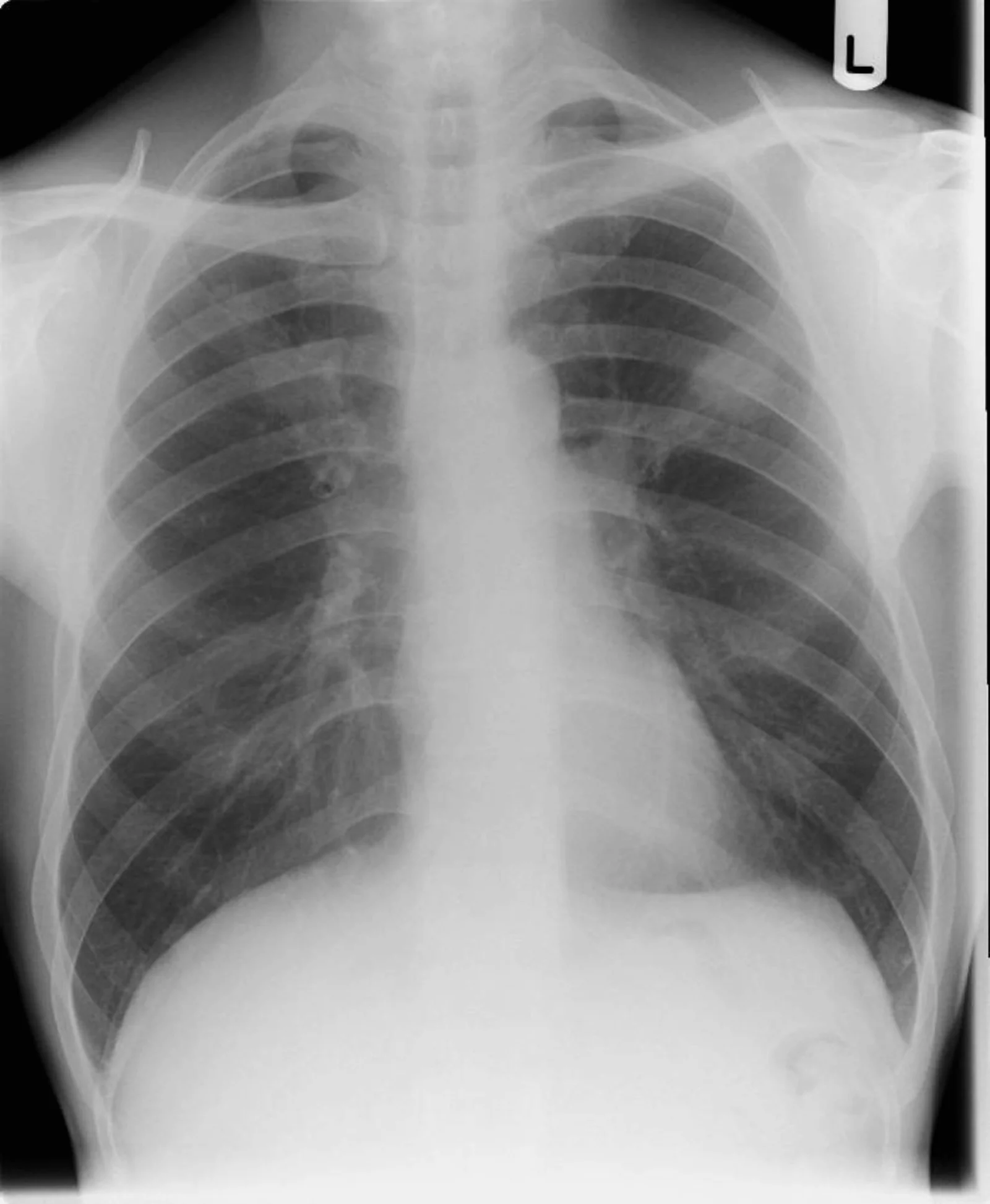
Posteroanterior chest radiograph The image is demonstrating bilateral ill-defined subpleural opacities without pleural effusion.

CT Chest confirmed multiple bilateral pulmonary nodules ranging from 1.2 to 3.8 cm, with a cavitating lesion in the right lower lobe and associated peripheral ground-glass halo (Figure 2).

**Figure 2:**
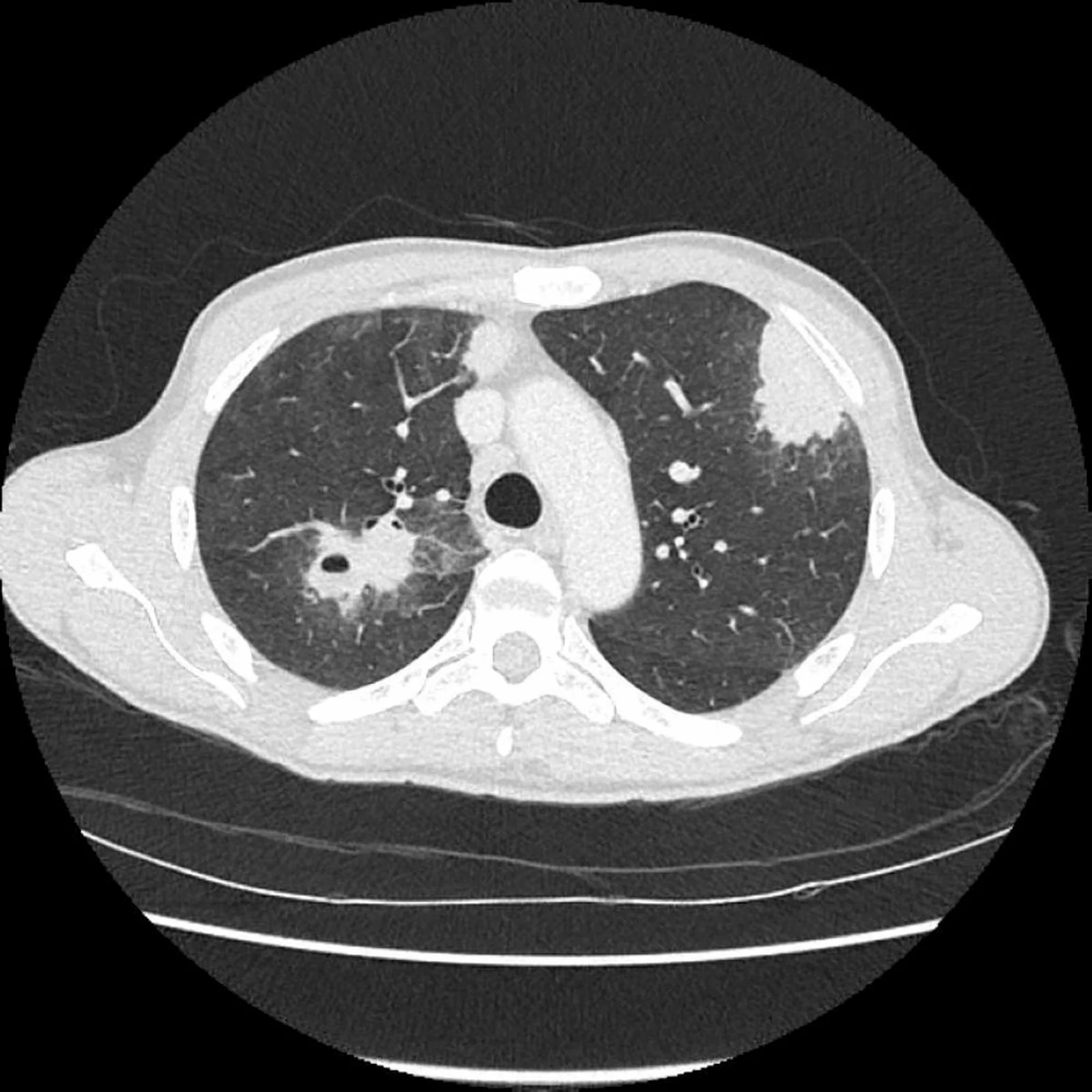
Axial CT chest The image is demonstrating bilateral pulmonary nodules of varying sizes with a cavitating lesion in the right lower lobe and associated peripheral ground-glass opacity.

On PET-CT, FDG uptake was intense, with an SUVmax of 20.1, a value exceeding that typically encountered in most inflammatory conditions and approaching the upper range documented in aggressive malignancies. Mediastinal lymphadenopathy was present across multiple stations. There was no evidence of extrathoracic hypermetabolic lesions on whole-body imaging. The combination of systemic symptoms, digital clubbing, cavitating and PET-avid bilateral pulmonary nodules with mediastinal lymphadenopathy established primary pulmonary malignancy (specifically squamous cell carcinoma or lymphoma) as the leading differential at this stage.

Bronchoscopy and tissue diagnosis

Bronchial washings demonstrated acute inflammatory changes with a sparse population of atypical cells. In the right lower lobe sample, these atypical cells were considered concerning for malignancy, particularly squamous cell carcinoma; however, a definitive diagnosis could not be established. Left upper lobe washings showed reactive changes with a single atypical cell of uncertain significance. Multidisciplinary team discussion and further tissue sampling were recommended. CT-guided percutaneous core needle biopsy of the largest pulmonary nodule (3.8 cm, right upper lobe) was performed, yielding adequate tissue for histopathological and immunohistochemical analysis. Given the cytological atypia and radiological features, there remained significant clinical concern for underlying malignancy, necessitating further tissue sampling for definitive diagnosis.

Haematoxylin and eosin (H&E) staining demonstrated a dense lymphoplasmacytic infiltrate with prominent admixed eosinophils. Storiform (cartwheel-pattern) fibrosis was present throughout the specimen. Importantly, obliterative phlebitis was identified, characterised by dense inflammatory infiltration of venous walls with luminal occlusion. There was no necrotising granulomatous inflammation, necrosis, or cytological atypia to suggest granulomatosis with polyangiitis (GPA) or malignancy, respectively. Immunohistochemistry did not demonstrate an increased number of IgG4-positive plasma cells, and the IgG4/IgG ratio was not elevated; therefore, formal histopathological criteria for IgG4-related disease were not fulfilled [6]. The histopathological findings are summarised in Table 2.

**Table 2:** Histopathological and immunohistochemical findings IgG4: Immunoglobulin G4; IgG4-RD: Immunoglobulin G4-related disease.

Feature	Finding
Storiform fibrosis	Present
Dense lymphoplasmacytic infiltrate	Present
Prominent eosinophilic infiltration	Present
Obliterative phlebitis	Present
Necrotising vasculitis	Absent
Necrotising granulomatous inflammation	Absent
Cytological atypia/malignant cells	Absent
IgG4-positive plasma cells per hpf	Not increased (did not meet threshold ≥10/hpf for lung)
IgG4/IgG ratio	Not elevated (did not meet threshold ≥40%)
IgG4-RD histopathological criteria met	No

Treatment and outcome

Written informed consent for publication of this case, including all accompanying clinical images, was obtained from the patient (see Disclosures). A diagnosis of probable IgG4-related lung disease was established on the basis of compatible clinical, serological, and histopathological findings. The ACR/EULAR 2019 classification criteria for IgG4-RD were applied; however, in the absence of diagnostic immunohistochemical findings, the case was considered most consistent with a probable diagnosis [5]. Cross-sectional imaging of the abdomen and pelvis, pancreatic protocol CT, and ophthalmological assessment were arranged to evaluate for extrapulmonary IgG4-RD involvement; these investigations were unremarkable.

Oral prednisolone was commenced at 0.6 mg/kg/day (40 mg/day). Given the cumulative steroid exposure anticipated, bone protection with calcium, vitamin D supplementation, and a bisphosphonate was initiated at treatment outset. Proton pump inhibitor gastroprotection was co-prescribed. Following four weeks at the induction dose, the prednisolone was tapered by 5 mg every two weeks, aiming for a maintenance dose of 5 mg/day at approximately six months, consistent with published management guidance [7,8]. The patient was reviewed at two-weekly intervals during induction and monthly thereafter.

At the three-month review, repeat CT thorax demonstrated significant resolution of bilateral subpleural opacities and a reduction in pulmonary nodule size by >50%. Serum IgG4 had fallen from 2.23 g/L at baseline, consistent with treatment response. At six-month review, the patient was asymptomatic with no exertional limitation. Repeat CT showed near-complete resolution of parenchymal opacities with residual stable small nodules and no new lesions. Mediastinal lymphadenopathy had resolved. The patient was maintained on prednisolone 5 mg/day with no clinical or radiological evidence of relapse at last follow-up.

## Discussion

Diagnostic complexity and initial diagnostic challenge

IgG4-related lung disease poses one of the most challenging diagnostic scenarios in thoracic medicine because its clinical, radiological, and metabolic features substantially overlap with primary pulmonary malignancy, lymphoma, sarcoidosis, GPA, and COP. The initial clinical presentation of this patient, including significant weight loss, bilateral cavitating pulmonary nodules, mediastinal lymphadenopathy, and an SUVmax of 20.1 on PET-CT, was highly suggestive of malignancy, and this differential appropriately dominated the clinical reasoning. An additional layer of diagnostic complexity in this case arose from cytological findings on bronchial washings, which demonstrated atypical cells concerning for malignancy. Although not definitive, such findings further reinforced the initial clinical suspicion of lung cancer and highlight the limitations of cytology in distinguishing inflammatory from malignant processes in IgG4-related disease.

An SUVmax of 20.1 is exceptionally high. While PET-CT avidity is well recognised in IgG4-RD and can equal or exceed values seen in malignancy, SUVmax values in this range are unusual even within the IgG4-RD literature and have been documented only in isolated reports [9,10]. This case reinforces the critical principle that FDG-PET avidity cannot reliably discriminate between inflammatory and malignant pathology, and that tissue diagnosis is mandatory in any equivocal case, irrespective of metabolic signal intensity. This case highlights the limitations of strict reliance on immunohistochemical thresholds in isolation and underscores the importance of clinicopathological correlation.

Atypical features requiring clinicopathological discussion

Two features in this case are not typical of IgG4-RD and merit explicit discussion: digital clubbing and cavitating pulmonary nodules. Digital clubbing is not a recognised manifestation of IgG4-RD in published case series or reviews [3,4]. Its presence in this patient raises two possibilities: first, it may be an incidental finding related to the former smoking history; second, it could represent a concurrent or alternative diagnosis. Because the biopsy did not demonstrate features of malignancy and was suggestive of a fibroinflammatory process, the most parsimonious interpretation is that the clubbing is smoking-related. However, this should be explicitly documented as a limitation rather than attributed to IgG4-RD.

Cavitation is also atypical for IgG4-RD, which typically produces solid nodules or mass-like consolidations [3]. Cavitating nodules raise a strong differential for GPA, mycobacterial infection, and squamous cell carcinoma. GPA was appropriately excluded by the combination of negative ANCA testing and the absence of necrotising granulomatous inflammation and necrotising vasculitis on histopathology. Infection was excluded by negative microbiological cultures. The cavitation in this case is therefore most likely attributable to the underlying fibroinflammatory process, though the precise mechanism (whether through ischaemic necrosis related to obliterative phlebitis or another pathway) remains unclear and warrants documentation.

Histopathological diagnosis and the role of obliterative phlebitis

The diagnostic evaluation in this case relied heavily on histopathology. The three cardinal histopathological features of IgG4-RD are storiform fibrosis, obliterative phlebitis, and dense lymphoplasmacytic infiltration with IgG4-enriched plasma cells [6]. In this case, storiform fibrosis, obliterative phlebitis, and a dense lymphoplasmacytic infiltrate were identified, providing strong morphological support for an IgG4-related fibroinflammatory process; however, immunohistochemical criteria were not fulfilled (Table 2). The presence of obliterative phlebitis is of particular diagnostic significance: it is highly characteristic of IgG4-RD and, together with storiform fibrosis, helps distinguish IgG4-RD from other major inflammatory differentials.

Quantitative immunohistochemistry is not optional in IgG4-RD diagnosis. Per consensus guidelines, lung tissue must demonstrate ≥10 IgG4-positive plasma cells per high-power field, and the IgG4/IgG ratio must exceed 40% [6]. Elevated serum IgG4 alone is insufficient: serum IgG4 may be normal in up to 30% of histologically confirmed cases, and conversely, it may be modestly elevated in malignancy, infection, and other inflammatory conditions [11]. In this case, the serum IgG4 of 2.23 g/L represents 1.65X the upper limit of normal (Table 1), a modest elevation that alone would not be diagnostically sufficient.

The neutrophilic leukocytosis: an unexplained feature

IgG4-RD is characteristically associated with eosinophilia rather than neutrophilic leukocytosis [2,12]. The leukocytosis of 16.2 × 10⁹/L with neutrophilic predominance observed in this patient is not typical of IgG4-RD and was not explained within the case. Possible explanations include concurrent occult infection, a corticosteroid effect (if commenced prior to the blood draw), or a physiological stress response. The peripheral eosinophil count, which is not reported, would have been informative. Future reporting of similar cases should include a full differential white cell count.

Differential diagnosis: systematic exclusion

The differential diagnosis in this case was methodically narrowed through a combination of serological, microbiological, and histopathological investigation. Primary pulmonary malignancy was excluded by the absence of cytological atypia or malignant cells on biopsy. GPA was excluded by negative ANCA testing (both PR3 and MPO) and the histopathological absence of necrotising vasculitis and necrotising granulomatous inflammation; storiform fibrosis and obliterative phlebitis, which are not typical features of GPA, further supported an IgG4-related fibroinflammatory process [13]. COP was excluded by the presence of storiform fibrosis and obliterative phlebitis, which are not features of COP, and by the elevated serum IgG4. Sarcoidosis was excluded by the absence of non-caseating granulomata on biopsy and the normal serum ACE. The presence of cytological atypia on bronchial washings further complicated the diagnostic process, as reactive atypia in inflammatory conditions can mimic malignant cytological features.

Treatment approach and response

Oral corticosteroids remain the first-line treatment for IgG4-RD, with published guidelines recommending induction doses of 0.6-1.0 mg/kg/day for four weeks, followed by a structured taper [7,8]. The dose used in this case (0.6 mg/kg/day) is at the lower end of the recommended induction range and is appropriate given the absence of life-threatening organ involvement. The clinical and radiological response in this case was excellent and consistent with published outcomes data: corticosteroid response rates exceed 95% in treatment-naïve IgG4-RD [8].

The optimal duration of maintenance therapy and the threshold for introducing steroid-sparing agents remain areas of active investigation. Rituximab has demonstrated efficacy as both a steroid-sparing agent and in relapsing disease, and azathioprine or mycophenolate mofetil have been used as conventional immunosuppressants in this context [7,14]. Long-term follow-up including serial serum IgG4, clinical assessment, and periodic CT imaging is recommended to detect relapse, which occurs in 30-40% of patients with IgG4-RD [8].

Limitations

Several limitations should be acknowledged. Immunohistochemistry did not demonstrate increased IgG4-positive plasma cells or an elevated IgG4/IgG ratio; therefore, formal histopathological criteria for IgG4-related disease were not fulfilled, limiting diagnostic certainty. Although sputum cultures were negative for mycobacteria and fungi, biopsy tissue was not separately tested with AFB staining or mycobacterial culture, which would have provided more definitive exclusion of tuberculosis. Representative histopathological photomicrographs and follow-up CT images were unavailable for reproduction. The peripheral blood eosinophil count was not documented, and its interpretation may also have been affected by corticosteroid exposure. C-reactive protein (CRP) and follow-up serum IgG4 levels were unavailable. The aetiology of digital clubbing was not definitively established, and a cardiac contribution was not formally excluded with echocardiography. Bronchial washing cytology demonstrated atypical cells concerning for malignancy, although these findings were non-definitive and may have represented reactive changes. A dedicated patient-perspective account could not be obtained, as the patient was not available for re-contact beyond the documented clinical encounters. Finally, follow-up beyond six months is required to assess longer-term relapse risk.

## Conclusions

IgG4-related lung disease is a rare but important cause of PET-avid, cavitating pulmonary nodules that can be indistinguishable from primary malignancy on clinical and radiological grounds alone. Accurate diagnosis typically requires histopathological evaluation demonstrating the pathological triad of storiform fibrosis, obliterative phlebitis, and quantitatively enriched IgG4-positive plasma cells; however, cases with supportive morphological features but non-diagnostic immunohistochemistry may pose significant diagnostic challenges. Elevated serum IgG4 is a useful supporting finding but is neither sensitive nor specific enough to substitute for tissue diagnosis. Clinicians should be aware that atypical features (including cavitation, exceptionally high PET-CT avidity, and neutrophilic leukocytosis) may be encountered in IgG4-RLD and do not preclude the diagnosis.

Early corticosteroid treatment yields excellent clinical and radiological responses in the overwhelming majority of cases, and accurate recognition of IgG4-RLD can spare patients from unnecessary oncological treatment even in diagnostically uncertain scenarios. Prospective registry data and longer-term follow-up studies are needed to characterise optimal steroid-sparing strategies and define relapse predictors in pulmonary IgG4-RD.
